# A Framework to Advance Biomarker Development in the Diagnosis, Outcome Prediction, and Treatment of Traumatic Brain Injury

**DOI:** 10.1089/neu.2021.0099

**Published:** 2022-03-23

**Authors:** Elisabeth A Wilde, Ina-Beate Wanner, Kimbra Kenney, Jessica Gill, James R Stone, Seth Disner, Caroline Schnakers, Retsina Meyer, Eric M. Prager, Magali Haas, Andreas Jeromin

**Affiliations:** ^1^ TBI and Concussion Center, Department of Neurology, University of Utah School of Medicine, Salt Lake City, Utah, USA.; ^2^ George E Wahlen Veterans Affairs Salt Lake City Healthcare System, Salt Lake City, Utah, USA.; ^3^ H Ben Taub Department of Physical Medicine and Rehabilitation, Baylor College of Medicine, Houston, Texas, USA.; ^4^ Semel Institute for Neuroscience and Human Behavior, David Geffen School of Medicine University of California, Los Angeles, California, USA.; ^5^ Department of Neurology, Uniformed Services University of the Health Sciences, Bethesda, Maryland, USA.; ^6^ National Intrepid Center of Excellence, Walter Reed National Military Medical Center, Bethesda, Maryland, USA.; ^7^ Department of Nursing and Medicine, Johns Hopkins University, Baltimore, Maryland, USA.; ^8^ Department of Radiology and Medical Imaging, University of Virginia, Charlottesville, Virginia, USA.; ^9^ Minneapolis VA Health Care System, Minneapolis, Minnesota, USA.; ^10^ Department of Psychiatry and Behavioral Sciences, University of Minnesota Medical School, Minneapolis, Minnesota, USA.; ^11^ Research Institute, Casa Colina Hospital and Center for Healthcare, Pomona, California, USA.; ^12^ Delix Therapeutics, Inc., Boston, Massachusetts, USA.; ^13^ Cohen Veterans Bioscience, New York, New York, USA.

**Keywords:** biomarkers, diagnostic biomarkers, neuroimaging, neurophysiology, prognostic biomarkers, traumatic brain injury

## Abstract

Multi-modal biomarkers (e.g., imaging, blood-based, physiological) of unique traumatic brain injury (TBI) endophenotypes are necessary to guide the development of personalized and targeted therapies for TBI. Optimal biomarkers will be specific, sensitive, rapidly and easily accessed, minimally invasive, cost effective, and bidirectionally translatable for clinical and research use. For both uses, understanding how TBI biomarkers change over time is critical to reliably identify appropriate time windows for an intervention as the injury evolves. Biomarkers that enable researchers and clinicians to identify cellular injury and monitor clinical improvement, inflection, arrest, or deterioration in a patient's clinical trajectory are needed for precision healthcare. Prognostic biomarkers that reliably predict outcomes and recovery windows to assess neurodegenerative change and guide decisions for return to play or duty are also important. TBI biomarkers that fill these needs will transform clinical practice and could reduce the patient's risk for long-term symptoms and lasting deficits. This article summarizes biomarkers currently under investigation and outlines necessary steps to achieve short- and long-term goals, including how biomarkers can advance TBI treatment and improve care for patients with TBI.

## Introduction

Biomarkers are objective, reproducible, and quantifiable measures reflecting biological processes. Biomarkers of injury may convey pathophysiological information, serve as proxies for injury progression or treatment response, and guide clinical decision making. The Food and Drug Administration (FDA) has described a biomarker as “a defined characteristic that is measured as an indicator of normal biological processes, pathogenic processes, or responses to an exposure or intervention, including therapeutic interventions”^1^ (see Table 1 for ideal biomarker requirements). As such, traumatic brain injury (TBI) biomarkers can facilitate diagnosis, interpretation, and monitoring of the injury course and thus augment patient support, management, and recovery.

**Table 1.** Biomarker Requirements

Term	Definition
Sensitive	Able to correctly detect or identify true positives (e.g., correlate with severity of injury)
Specific	Able to detect true negatives
Selective	Linked to brain injury (type/stage) or unique endophenotype
Safe	Sensitive enough to guide clinical decisions and ideally non- or minimally invasive with few adverse effects
Well-characterized	Release, half-life, clearance, kinetics in biofluid dynamics
Reproducible	Able to be replicated independently and comparable with appropriate normative data
Operational	Inexpensive and able to be collected and interpreted in a clinical setting
Optimized	Specific context(s) of use

TBI is characterized by an evolving and often multi-faceted pathology with many simultaneous changes occurring over hours, days, weeks, and years following the insult. For this reason, biomarker-based diagnosis and prognosis need to be applied and interpreted in the context of this evolving neurotrauma pathology. Further, because of the complex heterogeneity of TBI, it is likely that the optimal tool for assessing TBI will involve multi-modal components; specifically, a panel of blood-based and physiological biomarkers coupled with advanced neuroimaging that are appropriately obtained at multiple time points. It is also important to understand how a biomarker can be used to advance treatment for chronic somatosensory, neuropsychiatric, and cognitive deficits post-TBI. As part of the Brain Trauma Blueprint, TBI State of the Science, this article provides a review of the current state of TBI biomarker evaluation and development and provides a framework of recommendations needed to fill current research gaps in biomarker development.

The following section outlines a framework of the steps required to develop multi-modal TBI biomarkers into useful tools for clinical application.

1. Establish validity and reliability of the biomarker (i.e., can it be accurately and reproducibly measured)
2. Determine the biomarker's window of use (i.e., when is it best measured in relation to the injury event or disease onset)
3. Demonstrate that the assay/marker is clinically and pre-clinically applicable (to improve therapy translation)
4. Show qualification (i.e., is the biomarker associated with the target end-point)
5. Select utilization (i.e., what is the context of use)
6. Understand the biological rationale for using the biomarker (i.e., the causal pathway in which the biomarker is positioned)
7. Establish that interventions show relevant effects on the biomarker prediction in patient outcome
8. Demonstrate reproducibility (i.e., consistency between a test data set and a confirmatory data set)
9. Achieve qualification for regulatory acceptance (i.e., going through the FDA qualification process)

## Biomarkers and Treatment Development

Individuals with TBI present a diverse range of symptoms (from persistent symptoms to full recovery) because of the multiple clinical endophenotypes and biological underpinnings of their injuries. Many efforts have focused on identifying common, specific, and well-defined injury characteristics associated with TBI, with an overriding goal of identifying biomarkers that closely align with injury characteristics.

Biomarkers have multiple uses: *diagnostic biomarkers* identify the presence of TBI, *prognostic biomarkers* inform about expected outcomes in injured individuals, and *predictive biomarkers* predict response to a specific therapy and can be used to monitor response to therapy. Biomarkers may support clinical risk analysis and decision making, and can be used to stratify patients into pathobiologically defined (endophenotype-guided) subpopulations in clinical trials. Biomarkers can serve to screen and identify patients who may expect an altered, delayed, or complicated recovery or who might later develop progressive neurobehavioral symptoms and deficits (e.g., cognitive decline) as they age. Using biomarkers to provide inclusion/exclusion criteria for stratifying patients for specific outcomes could help reduce confounders in randomized controlled trials (RCTs) and will facilitate the development of targeted interventions in TBI.

Biomarkers can assess treatment effectiveness by narrowly determining target engagement or broadly tracking progressive atrophy and neurodegeneration caused by brain cell injury or death. Biomarkers that can accurately quantify decreased functioning and reversible injury are essential to monitor patient status and severity in the acute and subacute periods, especially after mild TBI (mTBI). However, to date, objective TBI indicators are still not commonly used in clinical practice (particularly in the absence of focal lesions) and biomarker use for mTBI remains unrealized despite being an active area of research. Finally, predictive and pharmacodynamic biomarkers may serve as early end-points in clinical trials evaluating new therapies, making biomarkers relevant as surrogate end-points. Thus, successful clinical biomarkers will channel heterogeneity in the presentation of patients with TBI and optimize diagnosis and treatment. Importantly, each biomarker needs to show robustness, validity, and reliability within its specific context of use.

For each of the mentioned biomarker use-category, biomarker examples include:

- Neuroimaging biomarkers, such as computed tomography (CT), structural magnetic resonance imaging (sMRI), functional MRI (fMRI), perfusion weighted imaging (PWI), magnetic resonance spectroscopy (MRS), positron emissions tomography (PET), magnetization transfer imaging (MTI), arterial spin labeling (ASL), near-infrared spectroscopy (NIRS), and single photon emission computed tomography (SPECT)
- Neurophysiological biomarkers, such as electroencephalography (EEG), magnetoencephalography (MEG), and eye-tracking
- Biofluid biomarkers, such as those from cerebrospinal fluid (CSF), saliva, sweat, urine, blood, and blood fractions, and include genetic, proteomic, and other marker classes
- Digital biomarkers, such as device-based readouts and wearables

The following sections provide examples of individual biomarkers that demonstrate how these tools may augment clinical trials and advance injury-alleviating treatments.

## Current State of TBI Biomarker Evaluation and Development

Biomarker development in the field of TBI is less developed than those in fields of oncology, cardiovascular disease, stroke, and some neurodegenerative disorders such as Parkinson's disease or multiple sclerosis. Progress in TBI biomarkers has been hampered by injury heterogeneity and limited availability of large, systematic observational studies that longitudinally (hours to years) collect and analyze multi-modal candidate biomarkers throughout the course of injury. Consequently, despite many peer-reviewed publications of candidate TBI biomarkers, most studies report small, cross-sectional cohorts and are not yet independently validated in larger, well-defined cohorts to determine clinical use. Nearly all biomarkers reported do not reach Level 1 Evidence (highest quality of methodology), which requires data obtained in a well-designed prospective RCT, meta-analysis of RCTs, or rigorous testing of previously developed diagnostic criteria. Rather, evidence for the use of imaging as a biomarker, for example, is generally Level II-III (prospective or retrospective cohort studies). To date, neuroimaging biomarkers are best established clinically for acute moderate to severe TBI (e.g., non-contrast head CT has Level I recommendation as a test for acute TBI and when there are signs of neurological deterioration), but aside from non-contrast head CT, most other imaging modalities are considered Level II-III (e.g., diffusion imaging).^2^ Advanced neuroimaging approaches, including MRI-based and PET-based modalities, provide insight into microstructural, functional, and physiological changes following TBI. However, these may require both large-scale normative data and FDA-approved quantitative diagnostic standards for clinical use. As such, many advanced neuroimaging approaches remain confined to the research setting. Likewise, physiological biomarkers hold great promise but are early in their development.

## Diagnostic Biomarkers

Using diagnostic TBI biomarkers for inclusion and exclusion of participants in RCTs will be a critical enhancement for clinical trials. Currently, many studies, particularly those including mTBI, are limited by the subjective nature of current diagnostic criteria that rely on clinical symptoms. Until objective candidate diagnostic biomarkers are validated, often subjective or inadequate clinical symptoms remain the status quo for diagnosis. Besides distinguishing between those with and without TBI, diagnostic biomarkers may also allow clinicians to distinguish patients based on their TBI endophenotype, and subsequently identify patients who may benefit most from specific interventions. Additionally, biomarker measures obtained prior to, during, and at the conclusion of an intervention can be used to assess treatment efficacy and classification accuracy. In the next section we summarize the use of biomarkers for diagnostic indications.

### Neuroimaging diagnostic biomarkers

Several imaging modalities are already used for clinical diagnosis and management. In the acute period after injury, clinicians use CT scans to identify life-threatening conditions that may require urgent treatment or surgical intervention, such as fractures, presence of foreign objects, bleeding, swelling, and distortion of the brain caused by space-occupying lesions. CT scans are available in smaller clinics and hospitals, use is standardized, and there are established guidelines for diagnosing TBI with CT.^3^ In the acute (up to 1 week) and subacute (between 1 week and 3 months) post-injury periods, clinicians may use routine MRI when the patient exhibits persistent or worsening symptoms. Routine brain MRI often includes sequences that provide enhanced anatomic detail compared with CT, which can provide clinicians with greater diagnostic clarity. Susceptibility artifact-sensitive sequences (e.g., susceptibility-weighted imaging) are also routinely available and provide information concerning the presence of microhemorrhage within the brain that is less visible on more conventional T2 sequences.^4^ Given that white matter microhemorrhage and diffuse axonal injury may often coexist, this readily accessible approach may provide additional diagnostic information on patients who are symptomatic but have a normal CT. However, given the lower frequency of these findings in mTBI and their inconsistent relationship to functional outcome, additional investigation is warranted before implementing changes in current clinical practice guidelines.^5–8^ In addition to routine CT and MRI, advanced quantitative techniques demonstrate significant promise to better characterize TBI in the acute, subacute, and chronic phases, and their use in TBI and concussion has been the subject of several recent reviews,^9–14^ which specifically detail quantitative neuroimaging findings.

In both the short- and longer-term phases of recovery, diffusion imaging (e.g., diffusion tensor imaging [DTI], diffusion spectrum imaging, diffusion kurtosis imaging) and volumetric analysis of three-dimensional anatomic imaging may continue to provide insight into both gross and micro-structural changes following TBI. Even in mTBI, studies generally report abnormalities in diffusion imaging metrics across several regions, most commonly in the corpus callosum, corona radiata, internal capsule, cingulum bundle, and long association pathways, including the superior longitudinal fasciculus.^15^ In children, adolescents, and adults, there is some evidence of initially increased fractional anisotropy and decreased apparent diffusion coefficient or mean diffusivity in the acute to subacute recovery phase, although longitudinal studies do not necessarily suggest a consistent pattern of DTI changes over time.^15,16^ Additional longitudinal studies and studies of mTBI are needed to further explore the precise trajectory of change in diffusion metrics over the course of recovery.

MTI may also add sensitivity to MRI evaluation of patients with TBI.^17–21^ MTI examines the presence or absence of macromolecules – such as proteins and phospholipids – that coat axonal membranes or myelin sheaths in white matter. MTI has been used to infer the degree of myelin integrity and Wallerian degeneration, inflammation, and edema in various disease processes, including TBI. This imaging modality has been evaluated more recently in experimental models of TBI in relation to histologically verified myelin loss^22^ and axon integrity.^23^ However, reports of clinical utility in patients have been limited when compared with other neuroimaging modalities.

PWI, dynamic susceptibility contrast MRI (DSC-MRI)/dynamic contrast enhanced (DCE) imaging, and ASL techniques may demonstrate vascular changes following injury. DCE-MRI can be used to quantify cerebral hemodynamics and regional blood flow by measuring the tissue concentration time curve of an injected contrast agent (e.g., gadolinium or gadobenate dimeglumine). One study of 27 deployed service members with a history of mTBI reported clusters of decreased perfusion in the right anterior and middle cingulate gyrus, the left cerebellar hemisphere, and the left cuneus relative to a comparison group; these measures were correlated with cognitive measures and symptom reports.^24^ ASL is a non-invasive method of perfusion imaging with MRI that uses water in the blood as an intrinsic contrast agent by electromagnetically labeling it near a region of interest and deriving differences between the images acquired with and without labeling. Several studies have reported ASL-based findings in patients with mTBI, reflecting altered perfusion in regions such as the thalamus,^25^ cingulate,^26^ striatum, and frontal and occipital lobes.^27,28^ ASL has also been applied in more severe forms of TBI, with findings of altered global perfusion as well as pronounced changes in the thalami and posterior cingulate.^29^ Additionally, there is limited evidence that ASL may detect persistent and widespread perfusion changes in a chronic post-injury interval that relates to cognitive functioning.^30^ However, further studies are warranted to determine the potential utility of ASL, including which regions may be most vulnerable, which time points may be most informative, and the expected direction of change.^26^

Emerging research suggests that dysregulation of cerebral blood flow may contribute to concussion pathophysiology.^31^ A change in cerebral blood flow in response to a measured vasoactive stimulus is defined as cerebrovascular reactivity and can be measured by various imaging techniques including CT, MRI, PET/SPECT perfusion techniques, and transcranial Doppler (TCD). Cerebrovascular reactivity (CVR) imaging is readily used for diagnosing and managing many cerebrovascular diseases, but has only recently been studied in the context of concussion.^31^ Some work has begun to evaluate CVR as a neuroimaging biomarker of traumatic vascular injury in sports concussion^32,33^ and after moderate-severe TBI.^34^ The hope is that this method could augment TBI patient management by facilitating diagnosis, classification, and prognosis of recovery. Further work is required to adapt this technique to provide accurate, reliable, and reproducible neuroimaging-based measures of CVR and to correlate the imaging measures with specific outcomes.

NIRS is a technology based on the absorption by chromophores (such as oxygenated and deoxygenated hemoglobin and cytochrome oxidase) of near infrared light (700–100 nm) passing through tissue. The reflected light attenuation reflects regional cerebral oxygen saturation and the balance between oxygen delivery and saturation. This technique is used to monitor cerebral oxygenation, blood flow and perfusion, intracranial bleeding, and increased intracranial pressure, primarily after more severe TBI.^35–38^ Most NIRS studies monitor oxygenation and autoregulation, particularly early after TBI.^36^ Despite its promise, NIRS still lacks sufficient standardization to replace more invasive measurements.^39–41^ Differences in NIRS methodology and equipment complicate translation for more widespread clinical adoption. Further, although small sample studies demonstrate promising agreement between NIRS data and other measures of cerebral autoregulation, additional studies are warranted to show independent outcome prediction from NIRS.

MRS, fMRI, PET, and SPECT provide information concerning physiological and/or metabolic abnormalities. MRS involves acquisition of a signal from hydrogen protons not associated with water and has been used to demonstrate the relative presence of a variety of metabolites, including choline (Cho), creatine (Cr), glucose (Glu), lactate (Lac), and *N*-acetylaspartate (NAA). A recent meta-analysis of 36 studies using MRS in the context of TBI revealed decreased NAA/Cr ratios in patients with severe TBI compared with controls^42^; however, these alterations were not observed in patients with mTBI. Additionally, these metabolic changes only emerged in the subacute to chronic phase of injury and were not seen acutely. Similarly, Cho/Cr ratios were only seen in patients with severe TBI in the subacute to chronic phase post-injury. Emerging MR techniques use nuclei with weak signals (e.g., calcium, magnesium, carbon, phosphorus) to provide additional information about the post-traumatic metabolism and to give insights into well-established secondary injury mechanisms such as oxidative stress, excitotoxicity, inhibitory dysregulation, imbalances in essential metabolites, and inflammation.^43^

Blood oxygen level dependent (BOLD)-fMRI sequences infer neurological activity based on the oxygenation state of blood and the response to activity-related metabolic demands. These sequences have been used to identify regions of brain activation under both task-oriented conditions and in a resting state. Utilizing graph theory, researchers have used fMRI to establish patterns of connectivity between brain regions and describe how these connections are altered during both normal development and disease. In TBI, task-based fMRI studies have been performed and demonstrate alterations in brain activity across a number of cognitive tasks in TBI patients, including working memory, motor networks, sustained attention, executive function, and language processing.^44–48^ Additionally, altered connectivity has been demonstrated in the default mode network and other brain regions following TBI.^49–52^ However, the quality of the BOLD signal depends on an intact neurovascular structure, and TBI is known to result in alterations of brain vasculature.^53–57^ As such, fMRI studies in TBI should be interpreted with caution.

PET and SPECT are techniques that allow for the detection and anatomical localization of radioisotopes associated with biologically active radiopharmaceuticals administered through the vasculature. PET involves coincident detection of high-energy photons resulting from positron decay. As such, PET has improved signal to noise and spatial detection when compared with SPECT-based techniques. In TBI, much of the work conducted with PET, to date, involves the imaging of glucose metabolism using ^18^F-fluorodeoxyglucose. These studies have generally demonstrated reductions in glucose metabolism in multiple brain regions of TBI patients and, in one study, are reported to correlate to the level of consciousness at the time of PET.^58–61^ However, increased metabolism has been reported in pericontusional regions as well.^58^ More recently, markers of neuroinflammation have emerged and have been utilized in human studies of TBI, including in active and retired National Football League (NFL) athletes.^62–65^ Data demonstrate that contact sports leading to concussion or mTBI are associated with increased neuroinflammation. Further, receptor-specific tracers have demonstrated alterations of benzodiazepine and cholinergic receptor binding in TBI patients using ^11^C-flumazenile and [^11^C]-methylpiperidin-4-yl acetate (MP4A), respectively.^66,67^ In addition to the tracers described, radiopharmaceuticals revealing pathological correlates of long-term neurodegeneration have received considerable interest in the setting of TBI. Specifically, the amyloid imaging agent Pittsburgh Compound-B (^11^C-PiB) has been used in multiple studies of patients with TBI. These agents have demonstrated positive findings in some studies^68^ and mixed results in others.^69^ Perhaps of greater interest are studies evaluating recently available radiopharmaceuticals targeting abnormally phosphorylated paired helical filament tau following TBI. Given the pathological finding of abnormal tau aggregated in chronic traumatic encephalopathy (CTE) post-mortem examinations, there is significant interest in developing an *in vivo* biomarker for detecting this condition. To date, several studies are reporting tauopathy in individuals with chronic TBI utilizing tau imaging agents,^70–72^ including increased tau labeling in a series of 26 former NFL players compared with 31 controls.

Although these advanced quantitative techniques have improved our understanding of TBI, their role in diagnosis is still developing, and clinical platforms remain forthcoming to utilize these approaches in patient care. Additional obstacles include difficulties in (1) directly comparing quantitative metrics derived from different scanners or acquisition parameters, and (2) the lack of adequate normative data to enable harmonization across centers, the latter of which is being reconciled by the development of a Normative Neuroimaging Library^73^ for MRI. Finally, although many of these imaging modalities demonstrate statistical relationships with symptom reports, cognitive functioning, and other outcomes, the validation of these biomarkers for diagnostic use in concussion and mTBI is complicated by the lack of consistent diagnostic criteria and the existence of other objective indicators of injury. Additionally, anatomic heterogeneity in TBI complicates use of common regions of interest in which to focus imaging.

### Neurophysiological diagnostic biomarkers

EEG records the averaged excitatory and inhibitory post-synaptic potentials of cortical pyramidal neurons, which tend to oscillate at different frequency bands (e.g., delta, theta, alpha, beta, and gamma bands) and involve cortico-cortical and thalamocortical connections. Advantages of electrophysiology include being inexpensive and easily transportable. Additionally, this technique has a high temporal resolution and therefore provides complementary information to technologies such as MRI, which provide a high spatial resolution. Several electrophysiological techniques, described subsequently, have promise for detecting mTBI.

A clinical review of EEG usually involves subjective visual inspection of brain electrical activity, assessment of topography and frequencies, and detection of pathological features. Typically, EEG is used to monitor and diagnose epileptic seizures arising in patients with acute TBI. EEG dysfunctions (such as focal slowing) appear to be related to blood–brain barrier (BBB) breakdown.^74,75^ Changes observed in mTBI clinical EEG have been repeatedly reported as non-specific, have a low inter-rater agreement, and might be more useful when used with other approaches such as quantitative EEG and/or event-related potentials.^76,77^

Quantitative EEG (QEEG) is considered more robust than clinical EEG and given its digital form, involves statistical analyses of the raw signal in order to provide numerical results and relevant information on EEG data. QEEG changes appear sensitive to symptoms experienced from mTBI, particularly balance instability.^78^ Studies with large sample sizes find that QEEG is sensitive for detecting mTBI.^79,80^

The capacity of EEG determinants – such as coherence, phase, and amplitude difference – to discriminate between mild and severe acute TBI during the post-acute period is high (sensitivity of 95% and specificity of 97%),^80^ and these results have been cross-validated in a sample of ∼500 patients evaluated through the Veterans Administration (VA).^79,80^ Studies using power spectrum analyses generally show a decrease in alpha power and an increase in delta, beta, and theta bands. These findings vary in different studies, requiring standardization across sites to improve consistency,^79,80^ and additional consideration is required to appropriately account for confounds and overlap in populations prone to presentation of psychiatric disorders.^81^ Portable EEG devices have been developed for the assessment of mTBI in sideline testing (for athletes), in theater (for military), and in more traditional treatment settings such as the emergency department. Finally, researchers recently combined transcranial magnetic stimulation (TMS) with EEG to study connectivity changes post-TBI, which might offer a promising avenue for investigating the neural substrates of connectivity dysfunction and reorganization post-mTBI.^82^

Event-related potentials (ERPs) allow researchers to understand cognitive processes using time-locked stimuli. P300 is related to attention and working memory. Its amplitude is often related to the amount of attention required by a task, whereas its latency is related to the time required for stimulus categorization and discrimination.^83^ Various studies have investigated ERPs and found altered control of thought processes and emotional processing in mTBI individuals with post-traumatic stress disorder (PTSD).^84,85^ Indeed, studies found that P300 responses were significantly delayed in latency and lower in amplitude in response to angry faces,^84^ suggesting that these individuals have difficulty in the recognition of facial affect. Other studies showed similar results in response to affective pictures and suggested reduced attentional resources and dysregulation of top-down processing in these individuals.^84,85^ A reduction in the amplitude of the P300 (∼40% of symptomatic athletes) and increased P300 latencies have also been observed in athletes with a history of concussion.^86,87^ P300 amplitudes correlate with the severity of post-concussive symptoms more so than do factors such as number of concussions, time since last concussion, severity of injury, or loss of consciousness.^88,89^

MEG records magnetic fields produced by electrical cortical activity and has better temporal resolution than EEG. MEG shows potential for the diagnosis of mTBI and has revealed abnormal activity in the frontal, parietal, and temporal regions in patients with mTBI.^90^ In a recent study, MEG demonstrated sensitivity in detection of changes in individuals with subacute/chronic mTBI (identifying abnormal brain activity in 87% of mTBI patients in delta waves [1–4 Hz]).^91^ In general, MEG is relatively expensive to acquire, non-transportable, not widely available, requires specialized expertise for analysis, is sensitive to cortical but not subcortical changes, and has only been collected in small cohorts. However, the use of MEG in TBI has been an area of expanding interest.^92^

When using electrophysiology as a diagnostic method for individuals with possible exposure to mTBI, one should keep in mind the impact of common technical difficulties on interpreting data (such as electrical artifacts, electrode placement, skull defects, medication effects, and patient alertness). For this reason, it might be more appropriate to use such approaches (QEEG and ERPs) in conjunction with other techniques such as neuroimaging and biofluid markers.

Vision and oculomotor assessment using eye-tracking devices, Saccadometers, and electrooculography are also used to assess mTBI and concussion.^93–99^ This method correlates with concussion symptoms in children and adults and has promising utility as a rapid, objective, and non-invasive aid for diagnosis. Several oculomotor measures have been investigated for use as potential biomarkers of altered brain function after TBI, including metrics of fixation, smooth pursuit, saccades,^100^ and convergence.^101^ One study demonstrated that oculomotor assessment may be an indicator of decreased integrity of frontal white matter tracts and of altered attention and working memory functioning.^102^ Moreover, researchers have developed mobile eye-tracking devices, which could benefit future clinical research by capturing eye movements remotely. However, there is not yet firm consensus regarding which visuomotor metric is most sensitive to brain injury-related change.

### Biofluid diagnostic, genetic and epigenetic biomarkers

The majority of TBI biofluid biomarker research has focused on diagnostic blood biomarkers of acute TBI, within the first 24 h after injury. Few candidates have been identified for the diagnosis of subacute or chronic sequelae of TBI. Biomarker profiles over weeks and months post-TBI from large, well-characterized cohorts will facilitate our understanding of TBI progression and are discussed under “biomarker profiling for monitoring.” Candidate acute TBI biomarkers include markers of BBB integrity, neuroinflammation, and axonal, neuronal, astroglial, and vascular injury.

The first combination TBI biomarkers to receive FDA approval in acute TBI are glial fibrillary acidic protein (GFAP) and ubiquitin carboxyl terminal hydrolase L1 (UCH-L1) with the Banyan Brain Trauma Indicator (BTI^TM^).^103^ GFAP is an astroglial intermediate filament structural cytoskeleton protein that is released upon injury and cell death; after acute TBI, serum GFAP levels peak 20 h after injury.^104^ UCH-L1, a neuron-enriched enzyme involved in ubiquitin turnover, is detectable as early as 1 h after TBI, peaks at 8 h, and then declines slowly 48 h after injury.^31^ Research has shown that the BTI test has high sensitivity and negative predictive value for predicting traumatic intracranial injuries on head CT scan acutely after TBI, and for distinguishing CT-positive, more severely injured, from CT-negative, mTBI patients.^105^ In the Evaluation of Biomarkers of Traumatic Brain Injury (ALERT-TBI) trial, levels of GFAP and UCH-L1 combined reliably differentiated TBI with CT-detected lesions from TBI lacking CT-detectable intracranial lesions (sensitivity of 97.5% and specificity of 99.6%).^106^ The BTI test is not approved for the diagnosis of TBI; rather, its indication is to identify TBI patients with intracranial lesion that may require surgical intervention. GFAP is shown in several other large studies as a diagnostic biomarker of acute TBI with imaging abnormalities, including Transforming Research and Clinical Knowledge in TBI (TRACK-TBI).^107^ Head injury Serum Markers for Assessing Response to Trauma (HeadSMART),^108,109^ Collaborative European NeuroTrauma Effectiveness Research (CENTER-TBI),^110^ and the National Collegiate Athletic Association (NCAA) Concussion Assessment, Research and Education (CARE) Consortium.^111^

S100 calcium binding protein B (S100B), an abundant astrocyte and oligodendrocyte protein, has a high sensitivity for TBI, with higher levels associated with greater TBI severity and poorer outcomes.^112^ Despite widespread use in Europe as a TBI biomarker, its specificity is lower in polytrauma patients because extracranial sources and varying levels in young children limits its potential as an acute point-of-care biomarker.^113^ TRACK-TBI conducted a phase 1 cohort (*n* = 1409) analysis of GFAP and S100B levels to predict intracranial abnormalities at 24 h after injury across the full spectrum of TBI (Glasgow Coma Score [GCS] 3–15). Receiver operator characteristic (ROC) curves for predicting patients with positive CT scans after injury had significantly higher area under the curve (AUC) for GFAP and S100B (0.85 and 0.67, respectively) than for negative CT scans.^107^ CENTER-TBI analyzed six serum biomarkers (S100B, neuron specific enolase [NSE], GFAP, UCH-L1 neurofilament protein-light [NfL], and total tau [t-tau]) in their consortium (*n* = 2867). They found that GFAP predicted CT abnormalities with highest discrimination ability (AUC 0.89), better than any other single candidate protein or combination of biomarkers in their panel.^110^ Likewise, the NCAA-CARE Consortium tested a panel of four candidate biomarkers (GFAP, UCH-L1, NfL, and t-tau) in 366 athletes and found that a combination of GFAP and UCH-L1 yielded the highest diagnostic discrimination (AUC = 0.71) between acute sports concussion and contact sports controls.^111^

Evidence for the use of other candidate diagnostic biomarkers is emerging. Neurofilament proteins are the major intermediate filament cytoskeleton structures of neurons. Tau is an axonal microtubule-associated protein that is associated with thin, unmyelinated axons and is present throughout neurons. Both structural proteins are associated with acute neuronal fiber damage. However, tau has less central nervous system (CNS) specificity than NfL as tau is also released after polytrauma.^114^ CSF NfL levels show high sensitivity for acute neuronal injury, including in boxers with mTBI.^115^ NfL levels in serum correlate with their CSF levels (*r* = 0.71),^116^ and recent reports show elevation in acute TBI across the spectrum of severity.^116,117^ Higher plasma tau levels collected within the first 6 h after injury may be prognostic of prolonged recovery from acute sports concussion. Amyloid isoforms, including amyloid beta 40 and amyloid beta 42, are associated with axons and accumulate as early as 2–3 h after TBI as a result of injured axons.^118^ However, acute CSF levels of these proteins are increased only after severe and not after mild TBI, making them less broadly useful as diagnostic biomarkers. This may be because of the microstructural organization of neurons being remote to capillaries and vessels, whereas astrocytes directly contact blood vessels with their end feet. Additional diagnostic biomarker candidates with highly brain-enriched expression include astroglial injury-defined biomarkers such as brain lipid binding protein (BLBP) and aldolase C (ALDOC), which show faster and longer elevation in the CSF of severe TBI patients than GFAP, and are elevated in the plasma and serum of severe and mild TBI patients. These biomarkers are currently being validated in the HeadSMART cohort of mTBI patients.^119,120^ ALDOC and BLBP also outperformed GFAP in a swine model of mild to moderate spinal cord injury^120^ (discussed subsequently). A variety of other serum or plasma proteins have also been reported to be diagnostic in acute TBI, as has been recently reviewed.^121^

Emerging candidate biomarkers of BBB disruption, neuroinflammation, neurodegeneration (e.g., alpha-synuclein,^122^ t-tau^123^) and vascular injury (e.g., vascular endothelial growth factor [VEGF], von Willebrand Factor, and angiopoietin-1) can facilitate our understanding of the complex TBI pathophysiology and are therefore emerging candidate TBI biomarkers, although they lack brain-specific expression and need to be further explored in the acute and chronic phase of TBI. BBB disruption and neuroinflammatory responses are an acute part of severe TBI,^124^ but their markers seem unchanged in boxers with mTBI and in military personnel with blast exposures,^115^ suggesting that these proteins are less sensitive as diagnostic TBI biomarkers. Similarly, a cytokine panel of interleukin (IL)-6, IL-10, tumor necrosis factor (TNF)-alpha(α), and VEGF are also elevated in participants with acute TBI with positive CT or MRI findings, with a combined discriminatory power of 0.92^125^ versus a healthy control cohort. Analyzing candidate TBI biomarkers in large longitudinal data sets, with statistical modeling, or using new technologies that measure low abundance biomarkers,^123^ for example, will advance the field, as they can provide TBI diagnostic panels for use from the point of care to chronic outcomes (see also sections addressing monitoring biomarkers).

In a new, but growing field, researchers are investigating the presence of exosomes in blood that carry biomarkers that may be useful in tracking TBI. Exosomes are lipid-membrane-bound extracellular vesicles whose cargo is rich in microRNA (miRNA) and protein, sequestered from the cytoplasm of the cell of origin.^126^ Exosomes are continually secreted by all mammalian cells, healthy and diseased, and appear to have a wide range of biological functions, including cell-to-cell communication and signaling. Because of their lipid bilayer membrane, exosomes easily cross the BBB and are abundant in peripheral circulation, and their cell of origin can be identified by the proteins they carry on their membrane. Free circulating exosomal and salivary miRNAs gain attention in the TBI field as potential diagnostic and prognostic biomarkers.^127,128^ miRNAs regulate post-transcriptional gene expression, and can be found in blood, saliva, and urine, as well as within exosomes. Researchers have evaluated whether serum and salivary miRNAs can act as specific and sensitive biomarkers of mTBI. The authors identified a subset of serum and salivary miRNAs that predict TBI likelihood through associations with head impacts and functional measures. Many of these miRNAs are thought to alter processes relevant to TBI^129^; however, these data, while promising, have not yet been confirmed.

### Digital diagnostic biomarkers

Several sensor systems are under development that can measure the impact of concussive forces on an individual's head in real time to help diagnose a TBI in the field. For athletes, some of these products are designed to fit inside a headband, helmet, or mouthguard. However, although some head-impact sensors are commercially available, their clinical utility is limited because of error rates associated with individual impact measurements, low specificity in predicting injury, and limited harmonization of data and use with other monitoring devices.^130^

### Multi-modal diagnostic biomarkers

Perhaps one of the most promising future arenas in biomarker development includes the use of multi-modal indicators for the detection of injury. Within the realm of fluid biomarkers, neuroimaging in particular, some investigators reason that biomarker panels could improve imaging-based injury diagnosis, as each may probe injury in a unique, but complementary way.^110,131^ Moreover, given the complexity of the multiple secondary injury mechanisms in TBI to one extent or another, it may be helpful to test multiple sources to identify a specific injury profile and tailor treatment more effectively.

Investigations are underway to thoroughly examine the relationship and co-variance of various biofluid, electrophysiological, and imaging markers that may detect TBI-specific pathophysiology. Correlation between quantitative neuroimaging and fluid biomarkers^132^ or between neuroimaging and EEG are examples that illustrate multi-domain cross-validation of noninvasive assessment tools.^133^ Clinical utility demands that widely usable biomarkers be easy to administer, quickly interpretable, and cost effective. Multi-modal approaches will help to establish the most useful panel or series of tests that then should undergo the same rigor. Machine learning may also be beneficial for arriving at suitable combinations for most accurate diagnostics and prognostics; it can uncover meaningful relationships among multiple candidate biomarkers across domains.

## Prognostic and Predictive Biomarkers

Prognostic biomarkers in TBI can be divided into two categories: (1) those that are measured early after injury and predict evolving TBI sequelae, and (2) those that are measured in the chronic phases and predict long-term outcomes, such as the development of neurocognitive or movement disorders. Like diagnostic ability, prognostic capability of biomarkers can improve clinical studies by helping to stratify patients based on their injury phenotypes. Predictive biomarkers inform an individual's likelihood of responding to a treatment and could help in understanding clinical TBI treatment outcomes. In this way, biomarkers may augment the development and dose-response assessment of a drug or may help predict the success of an intervention prior to eventual outcomes. Thus, early, objective signs in form of modified biomarker profiles could expedite future clinical trial evaluation and may hence assist in optimizing patient treatment.

### Neuroimaging prognostic biomarkers

Use of neuroimaging for clinical prognosis is currently far more limited than for diagnosis. CT remains the most widely used method for determining prognosis following TBI, but it is confined primarily to patients with moderate to severe TBI. The Marshall classification system has been available since 1992 and classifies patients based on the presence of cerebral edema and midline shift, and the presence and size of intracranial hemorrhage.^134^ The Rotterdam scoring system is more recent and incorporates multiple injury types, including consideration of the presence of subarachnoid hemorrhage.^135^ Both systems have been utilized to predict mortality and outcome following moderate to severe TBI.^136–141^ At present, approaches of neuroimaging for prognosis are lacking for mTBI/concussion, though one meta-analysis revealed that lesion patterns (e.g., brainstem lesions, diffuse axonal injury [DAI] patterns) were associated with neurological outcome.^142^ Quantitative MRI could aid in predicting outcome in all severities of TBI and could presumably be used in the subacute phase to predict persistent or worsening symptoms. For example, a recent review of studies on mTBI that utilize diffusion imaging reported the extent to which diffusion metrics are associated with symptom reporting (e.g., anxiety, depression, pain, headache, light sensitivity, fatigue, or post-concussion symptoms more generally) and/or recovery duration. The majority of these studies concluded that a relationship between diffusion imaging findings and symptom presentation exists, whereas a minority reported no correlation. Similarly, the majority of studies found a relationship between diffusion imaging and cognitive performance (e.g., attention, memory, processing speed, executive functioning, mental status), particularly in the early recovery phase.^15^ Other techniques, described in more detail subsequently, are promising, but are also still in the discovery phase.

In addition to loss of neuronal elements and deficient connectivity, chronic neurodegenerative changes, such as CTE, are seen in patients with a history of TBI, particularly in the setting of repetitive injuries resulting from multiple impacts. CTE is considered a pathological diagnosis that can only definitively be made at autopsy. However, with the recent emergence of specific radiopharmaceuticals that may be imaged through PET, this condition could potentially be diagnosed through neuroimaging during a patient's lifetime.

Excess tau, and particularly abnormally phosphorylated tau (p-tau), are implicated as a biomarker of long-term negative consequences of brain injury and are associated with many neurodegenerative diseases. Researchers have investigated the use of PET/MRI to detect the uptake of a tau-binding tracer as a potential biomarker. Tracer uptake is shown to be associated with exposure to blast neurotrauma, but not with symptom duration or blunt neurotrauma.^143^ Recent work by Stern and coworkers demonstrates elevated tau levels in former athletes as measured by PET compared with controls.^72^ Although much of the work on tau currently exists as aggregate analyses, additional work examines whether tau neuroimaging is a viable diagnostic tool in the context of the individual patient. Future work may identify and validate other promising PET ligands for TBI.

Other recent neuroimaging techniques examined the role of inflammation in TBI. For example, one study focused on former NFL players and utilized a second-generation translocator protein (TSPO) radiopharmaceutical that can be imaged through PET and detects microglia activation during inflammation. Preliminary work using this method found that cognitive disruption in verbal learning and memory was associated with an increase in TSPO in specific brain regions, such as the supramarginal gyrus and right amygdala.^62,63^ With further validation in larger sample sizes, this technology could enable a less invasive, quantitative way to examine and predict post-traumatic brain changes after TBI. This could be applied to clinical trials that longitudinally study pathological responses to trauma or could be incorporated into prognostic evaluation and therapeutic monitoring.

Researchers are currently evaluating patterns of white matter hyperintensities viewed on MRI as a neuroimaging biomarker. Although these foci of abnormally increased signal intensity are routinely noted as incidental findings in neuroimaging, they have demonstrated associations with neuropsychological performance and fatigue in TBI.^144^ Additionally, they have shown prognostic value in both adult and pediatric TBI populations. In particular, lesion volume in the frontal lobe is reported to correlate with functional outcome and injury severity 1 year after injury^145^; however, another study failed to find a relationship between frontal lesions and functional outcomes.^146^ An obstacle to translating this approach into clinical practice and applying it to clinical trial study designs is that the manual identification of these lesions is time consuming and impractical. A recent study proposed an automated framework to quantify white matter hyperintensities in multi-modal MRI using random forest, a machine learning algorithm.^147^ Automated or semi-automated tools such as these, if shown to be specific and efficient, could be useful for mining large-scale data sets to determine the diagnostic or prognostic value in the context of TBI.

### Neurophysiological prognostic biomarkers

Beyond the acute and subacute stages, mTBI is often characterized by DAI. In parallel, changes in brain electrical activity following TBI can be persistent. One study reports that 85% of mTBI patients who presented significant EEG changes acutely continue to present altered EEG up to 1 year after injury.^148^ Several studies also show that EEG slowing from left temporal regions is associated with chronicity of post-concussion symptoms at the 6- and 12-month follow-up.^149,150^

Using QEEG, several studies showed changes in power spectrum (i.e., reduced alpha and theta frequency bands and an increased delta frequency band) over time,^148,151^ but a return to a normal range within 6 months post-concussion.^74,152^ Another group reported that QEEG phase (i.e., functional connectivity) can predict outcome of patients with mTBI (90% accuracy) at 1 year post-injury and demonstrated greater accuracy than CT and power spectrum.^153^ Two studies showed that P300 amplitude and latency of mTBI does not differ from the control group at 3 months to >2 years after injury,^154–156^ whereas another group found that ERPs can be altered in patients with mTBI >6 years after injury.^157^

Specific evidence related to mTBI prognosis using EEG methods is relatively sparse. Nevertheless, most findings suggest that QEEG may have utility for longitudinal evaluations, but confirmatory experiments are needed. One challenge of using EEG is its lack of specificity. It would likely be difficult to use EEG alone to distinguish between different mental states and their causes (e.g., TBI vs. depression); therefore, EEG could serve as a complement to other assessment measures.

Although currently limited, recent data suggest that TMS has prognostic value in detecting neurophysiological changes post-concussion.^158^ Indeed, at 1–5 years post-concussion, no differences are observed in the amplitude of motor-evoked potentials (MEP); however, an increased motor threshold (i.e., the lowest stimulus intensity to produce a detectable MEP) is found when compared with uninjured controls.^159,160^ Another study found a lengthened duration of the cortical silent period (cSP) (i.e., interruption of voluntary muscle contraction after TMS of the contralateral motor cortex) in concussed patients versus controls.^159,161,162^ Despite finding no intracortical facilitation differences among single concussion, multiple concussions, and control groups, studies have found that concussed patients have longer intracortical inhibition than controls.^161,162^ Two studies reported similar long-term neurophysiological changes (> 5 years post-concussion), such as a lengthened cSP of shorter duration and a longer intracortical inhibition in retired athletes than in controls.^161,163^ Therefore, long-term changes in intracortical inhibition, but also increased stimulation threshold and slowed neurological conduction time, may be useful indicators when considering prognosis of mTBI. Nevertheless, additional studies are needed to confirm the interest of TMS as a prognostic tool.

### Prognostic use of biofluid, genetic, and epigenetic biomarkers

There is a need for biomarkers to facilitate return-to-play/work/school/duty decisions for mTBI and to predict who may experience prolonged post-concussive symptoms. The use of prognostic biomarkers is less mature than that of diagnostic biomarkers. It is easier to validate short-term prognostic biomarkers because of their closer temporal association to the outcome of interest.

NfL is identified by several studies as a promising prognostic biomarker in TBI of all severities.^116,117,164,165^ Elevated levels of both plasma and exosomal NfL is associated with multiple (≥ 3) mTBIs and remote neurobehavioral symptoms in service members and veterans enrolled in the Chronic Effects of Neurotrauma Consortium (CENC) longitudinal study.^164^ Serum NfL correlates with persistent post-concussive symptoms with an AUC of 0.81 in Swedish hockey players.^116^ Among civilian TBI survivors (*n* = 230), serum NfL correlates with initial injury severity and 5-year functional outcomes as well as with imaging measures of atrophy and axonal injury documenting not only its predictive ability but also multimodal validation.^116^ Brain-derived exosomal NfL is associated with decreased cognitive function in elderly veterans with remote TBI symptoms.^165^

Tau also has promise as a prognostic biomarker. Studies have reported elevated plasma t-tau and p-tau, as well as a ratio of p-tau over tau in severe TBI patients 6–8 months after injury.^166^ T-tau in blood samples collected 1 h after sports-related concussion showed diagnostic accuracy for TBI.^167^ A recent study found that levels of tau, ß-amyloid-42, and IL-10 were higher in exosomes of military personnel who had experienced mTBIs than in personnel who had not.^168^ Among TBI patients, regression models show that post-concussive symptoms are most related to exosomal tau elevations, whereas exosomal IL-10 levels relate to PTSD symptoms. In the CENC cohort (*n* = 195), experiencing multiple (≥ 3) mTBIs is associated with increased exosomal t-tau and p-tau as well as with later neurobehavioral symptoms.^169^ Further prospective study designs are needed to examine these biomarkers’ longitudinal changes in and outside of brain-selective exosomes.

miRNAs have also gained attention in the TBI field as potential prognostic biomarkers.^127^ One study assessed the performance of a panel of serum miRNA biomarkers on indicators of concussion, subconcussive impacts, and neurocognitive function in collegiate football players over the span of the playing season.^126^ Athletes with declining neurocognitive function over the season showed corresponding increases in miRNA concentrations. Collegiate football players showed some miRNAs associated with baseline concussion assessments and with neurocognitive changes between pre- and post-season. Once those promising findings are validated, such miRNA biomarkers could serve to identify athletes at risk for declining neurocognitive status.

Genetic biomarkers can potentially predict risk and resilience to TBI as well as short-term versus long-term TBI symptom burden. Among individual genetic variants, the ɛ4 allele of the apolipoprotein E (APOE) gene, which supports lipid transport and injury repair in the brain, is most strongly associated with poorer outcomes after TBI. Individuals with the ɛ4 allele are 10 times more likely to develop dementia following a TBI. The ɛ4 allele has an increased risk of impaired verbal memory 6 months after mTBI.^170^ Other single nucleotide polymorphisms (SNPs) linked to TBI prognosis influence post-injury tau hyperphosphorylation, a key index of neurodegeneration.^171^ In animal models, transgenic mice carrying the human P301S mutation of the microtubule-associated protein tau gene have (20 times) greater tau hyperphosphorylation after 1 mTBI, which is exacerbated after repeated mTBIs, as hyperphosphorylation is 50 times greater after 4 mTBIs, and 60 times greater after 12 mTBIs.^172^ Researchers observed a similar mechanism for the Val66Met SNP of the brain-derived neurotrophic factor (BDNF) gene, which plays an important role in neuroplasticity.^173^ The BDNF Met66 allele is linked to decreased tau hyperphosphorylation,^174^ as well as better cognitive outcomes in the years following severe TBI in male Vietnam veterans.^175,176^ Importantly, the protective benefits of Met66 appear to be age dependent, conferring lower mortality and greater TBI-related protection for those >45 years of age but higher mortality risk for those < age 45.^177^ How and when these variants show their best prognostic potential awaits further investigation before they may benefit intervention trials.

To date, genetic research on TBI prognosis has not expanded beyond studies of individual SNPs except for a recently published Genome-Wide Association Study (GWAS) that identified two significant risk markers for concussion.^178^ GWAS meta-analyses, maximizing utilization of previously collected cohort data and biosamples, would help accelerate disease understanding, target identification, and risk assessment in this field, as has been the case for many other brain disorders.

In addition, several studies applied polygenic risk scoring (PRS)^179^ to determine if genome-wide measures of vulnerability for other, possibly related disorders or traits could be predict TBI prognosis. In a sample of United States service members, a PRS related to infant head circumference is significantly associated with progressive cognitive/emotional post-concussive symptoms.^180^ Similarly, higher PRS scores for Alzheimer's disease (reflecting greater genetic risk) is linked to increased cortical thinning and reduced memory performance in veterans with remote history of mTBI.^181^ These results serve as a proof of concept that genetic biomarkers can shed light on TBI prognosis, although optimal usage of PRS-derived risk grouping needs to be further developed.

Another potential prognostic biomarker for a unique TBI endophenotype is chronic pituitary dysfunction, which may occur in 30–80% of patients 2–3 years after injury,^182^ but data are limited and primarily based on case series. However, to link malfunctioning of this system to TBI is challenging, as neuroendocrine dysfunction is usually not diagnosed until 5–10 years later. The most commonly affected pituitary hormone is growth hormone, which is disrupted in up to 25% of patients with TBI. Currently, diagnostic tests to identify acquired growth hormone deficiency are limited to the insulin tolerance test in the United States, which requires a specialized endocrinology clinic for assessment. Abnormal pituitary hormone levels correlate with long-term cognitive symptoms. These hormone disturbances are often treatable by hormone replacement therapy that can improve cognitive function and overall well-being in those who are identified, making this an attractive clinical direction. Early studies explore growth hormone, adrenocorticotropin, and pituitary hormone in the context of TBI, and examine their use in TBI management.

## Biomarker Profiling for Monitoring

**FIG. 1.**
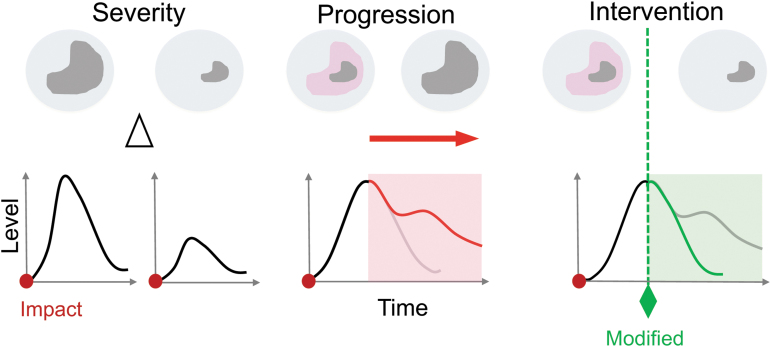
Profiling traumatic brain injuries (TBIs) with a corresponding biomarker response. The schematic depicts three different brain injury states (top) that associate with typical fluid biomarker profiles (bottom) to assess patient status/trajectories or specific pathophysiological processes after the onset of injury (red dot). **(1)** Severity: a severe (top left) or mild (top right) brain injury might lead to a lesion (gray). An accompanying biomarker profile (bottom) should strongly correlate with differences (delta) in the severity of the injury, with higher elevations of a biomarker expressed as the severity of injury increases. **(2)** Progression: acute TBIs cause primary injuries that result in irreversible neurodegeneration (gray). Over time (hours to weeks) the primary injury triggers progressive secondary biochemical cascades in perilesional, compromising tissue areas (pink). Biomarker trajectories profile the temporal injury progression with an increase in release after the primary injury (initial peak; black line) and secondary injury profiles that correlate with injury progression (red line). **(3)** Intervention: biomarker trajectories should profile treatment interventions. A treatment intervention at any time (dotted green line) may ameliorate injury progression and reduce secondary injury. The biomarker trajectory may subside (green line) in response to the treatment. Color image is available online.

**FIG. 2.**
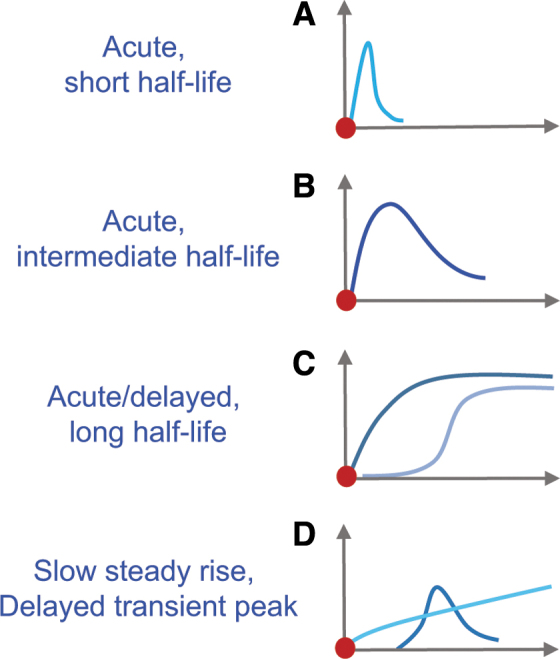
Biomarker kinetics in biofluids. Variations in biomarker release, stability, proteolytic breakdown, and clearance in circulation contribute to differences in their fluid profile. Varying profiles result from different release kinetics (e.g., membrane poration, cell death [necrosis]), and different half-lifes depending on size and vulnerability to proteases. **(A)** Acute, short half-life: biomarkers (e.g., S100 calcium binding protein B [S100B], ubiquitin carboxyl terminal hydrolase L1 [UCH-L1], fatty acid binding protein 7 [FABP7]/brain lipid binding protein [BLBP]) show an acute peak followed by rapid clearance within minutes to hours. These biomarkers may be used to detect individual insults. **(B)** Acute, intermediate half-life: biomarker (e.g., glial fibrillary acidic protein [GFAP], neuron specific enolase [NSE]) rise acutely and remain elevated for hours to days, before clearance. These biomarkers might be suitable for diagnostic purposes. **(C)** Acute/delayed, long half-life: stable biomarkers (e.g., aldolase C [ALDOC], spectrin breakdown products [SBDPs]) with an acute or delayed rise and maintained elevation for days to weeks. These biomarkers are suited for acute and chronic diagnosis. **(D)** Slow steady rise, delayed transient peak: biomarkers (small GFAP-breakdown products [BDPs], myelin basic protein [MBP], tau/phospho-tau) show a slow and steady increase in expression over time (weeks to months) or a delayed, transient or steady elevation. These biomarkers might track ongoing tissue atrophy or chronic sequelae. Color image is available online.

Biomarker trajectories can monitor a patient's status or specific pathophysiological processes over time and potentially even provide safety readouts of possible toxicity or side effects of an intervention in clinical TBI trials (Fig. 1). Profiles of biomarkers sensitive to evolving TBI processes may benefit treatment monitoring as predictive or pharmacodynamic indicators (e.g., a measure of attenuated inflammation, reduced fiber tract atrophy, or neuronal plasticity). Biomarker trajectories alone or as a panel of several different markers may be combined to serve as monitoring biomarkers tracking patient progression (e.g., imaging, biofluid, physiological). For this context of use, it is critical to determine biomarker profiles and half-lives in circulation to optimize each marker's use over time to most effectively monitor TBI patients’ progression (Fig. 2).

### Neuroimaging monitoring biomarkers of injury progression

TBI can inflict acute irreversible brain damage in the form of neuronal/glial cell death, traumatic axonal injury, and vascular injury. TBI also results in neurological deficits caused by metabolic depression, edema, excitotoxicity, and ionic dysregulation.^183–185^ These pathophysiological injury types occur secondary to the primary traumatic insult and likely compromise the function of the neurovascular unit (NVU), which integrates the relationships among capillaries, neuronal networks, and glia.^186^ Imaging modalities such as MRI, CT, PET, SPECT, or TCD can show alterations in blood flow and/or hyperemia and may help to identify perilesional or pericontusional tissues “at risk.”^43,187^ MRI can be used as a tool for identifying regions of the brain that have incurred injury; however, it requires standardization and calibration of instruments to monitor brain health over time, normalization across scanners and recording vocabulary, and deep machine learning algorithms to associate imaging features and clinical phenotypes.

Contrast-enhanced neuroimaging administered in TBI patients may help to identify BBB disruption and associated vasogenic edema. Alternatively, diffusion-sensitive techniques such as diffusion weighted imaging with calculation of apparent diffusion coefficient maps may identify restricted diffusion, thereby identifying areas of cytotoxic edema and active necrosis. In the setting of contusion or in brain regions where blood flow is compromised, tissue around the central area of injury may be at risk of deteriorating over time. As such, it is called perifocal, pericontusional, or perilesional tissue with blood flow and energy metabolite changes.^188,189^ Metabolic depression is not only present after severe TBI but is also a major endopheotype of mTBI and concussion.^190^ The concept of metabolic vulnerability is broadly accepted as an important mechanism of TBI progression and is a contributor to exacerbated symptoms after repeated mTBI.^191^ In a small cohort of concussed football players, ASL monitored decreased cerebral blood flow that recovered over various time periods and was associated with psychological symptom perseverance.^192^ Microdialysate measures document a low oxygen extraction fraction, decreased oxygen/glucose ratio, and increased lactate/pyruvate ratio in TBI patients.^185^ Additionally, scientists have recently adapted pH-weighted molecular MRI to monitor metabolic vulnerability caused by secondary metabolic and ion imbalance-related traumatic injury processes.^193^ Chemical exchange saturation transfer imaging monitors cerebral acidosis secondary to TBI and shows promising correlations with recovery scores using the Glasgow Outcome Scale – Extended (GOS-E) at 6 months post-injury. This pioneering work bridges the acute pathophysiology of at-risk tissue with accepted outcome measures and provides images of abnormal brain physiology as a consequence of TBI.

Perilesional tissue is a prime target for TBI therapies, as it is potentially salvageable and, therefore, imaging and metabolism-related biomarkers may be useful as near-term predictive and pharmacodynamic biomarkers. Versions of nuclear spin magnetic resonance spectroscopy are promising imaging modalities to track the energy state of the injured brain and follow the metabolic recovery after concussion. These techniques use metabolites like NAA and other compounds (H^1^, P^ 31^, C^13^-MRS)^194–196^; however, confirmation of selectivity is needed. Metabolic profiling monitors impaired bioenergetic state after mTBI using gas chromatography mass spectrometry.^184,197,198^ However, such metabolomic screens need to rigorously assess brain specificity for compounds to be developed into clinically useful mTBI monitoring tools. Brain-specific metabolic protein biomarkers such as astroglial metabolic proteins with brain specificity can inform about brain tissue compromise and metabolic crisis.^119^

### Neurophysiological monitoring biomarkers

Clinicians typically use EEG in the acute stage to monitor seizures in TBI patients, but have also used this tool to detect changes in acute mTBI over time (months to years).^199^ Monitoring brain activity using EEG can provide insight into the status of minimally conscious patients with TBI.^200^ This study showed that EEG measures of behavioral states provide distinctive signatures that complement behavioral assessments of patients with hemorrhage shortly after TBI. More research is needed to link these measures to patient outcomes.

### Biofluid monitoring of genetic and epigenetic, actively secreted and trauma-released biomarkers

To successfully employ biofluid markers as TBI monitoring tools, it is critical to comprehend biomarker trajectory, including cellular release appearance in CSF and blood as well as degradation and clearance after injury (Fig. 2). The temporal profiles of GFAP, UCH-L1, and S100B are partially described, yet correlations to underlying pathophysiological processes leading to their temporal profiles are still largely elusive.^112,126,201^ A recent pilot study shows that the percent change in serum UCH-L1 and S100B discriminated between concussed and non-concussed athletes, whereas levels at individual time points did not.^202^ Monitoring percent change, accomplished by either comparing individuals’ change to pre-injury or pre-season levels, reduces noise because of large inter-individual heterogeneity. However, most emergency care providers typically do not have patients’ individual baseline biomarker levels as they present with mTBI. Therefore, in addition to relying on generalized reference values, careful monitoring of repeated measurements can provide a patient's rate of change, allowing for individualized profiling during acute short-term care.

Measuring S100B levels in serum – and more recently in saliva – is a useful marker for assessing brain tissue after TBI.^203,204^ However, S100B lacks specificity as it is elevated following orthopedic trauma outside the brain and hence has limited its use in polytrauma patients^205^; S100B is therefore not used widely in North America. However, clinicians in Europe have monitored S100B to evaluate mTBI patients’ need for a head CT and to detect secondary injury progression.^112^ Continually elevated t-tau levels in preliminary studies of concussed athletes are associated with persistent post-concussive symptoms, whereas athletes with normal or only mildly elevated plasma tau resolve their symptoms and returned to full competition.^206^ Therefore, tau may be a potential biomarker to monitor recovery in athletes with TBI and could be used as a guide to allow them to safely return to play.

In addition to biofluid kinetics, differences in cell release provide insight into biomarker profiles. The brain-specific isoform of the glycolytic enzyme aldolase (ALDOC), is rapidly released from membrane-wounded astrocytes in a human stretch-injury model as well as in mouse, swine, and rat neurotrauma models. The *in vivo* models document depleted astrocytes in pericontused regions.^119,207^ Proteomic studies in these trauma models as well as in clinical studies demonstrate release of ALDOC along with many other metabolic enzymes in the early hours after injury to the cells.^119,207^ The same studies show that ALDOC levels remains elevated for days after TBI. Despite our limited knowledge on biomarker kinetics, these examples show uses of monitoring biomarkers to track progression of TBI-related injury, outcome prediction, and contribution to personalized patient care (Fig. 2).

### Digital monitoring biomarkers

Technological development in the wearable device and remote sensor field could facilitate objective TBI symptom monitoring. For example, advances in portable EEG monitoring, sleep assessment, gait analysis, cognitive function testing, eye tracking, and voice analysis could provide remote tracking outside of clinical confines. In particular, sleep parameters measured by wrist actigraphy are already being used as endpoints for clinical trials. Other emerging technologies will need further testing for validity and reliability before being used in clinical research.

## The Future of Precision Medicine: Multi-Modal Biomarkers and Biomarker Panels

TBI symptoms and pathophysiology vary from patient to patient and over time; therefore, a single biomarker is not sufficient for diagnosis, prognosis, or monitoring across the TBI spectrum. It is more likely that biomarker panels are needed to best assess the diverse clinical phenotypes and heterogeneous pathophysiology of patients with TBI, a challenge for the neurotrauma field in examining multiple biomarkers including markers from different modalities for effective joint benefit.

Although this field is in its infancy, a few studies have begun to explore combining biomarkers for TBI. One study evaluated a panel of blood-based biomarkers with and without neuroimaging findings (CT and MRI) and whether it discriminates between patients with suspected mTBI using single-molecule array technology.^208^ The panel included GFAP, tau, UCH-L1, and NfL. Combining GFAP, tau, and NfL showed satisfactory discriminatory power in relating to MRI-detected abnormalities, even in mTBI patients with a normal CT. This study highlights the potential of a multi-modal approach to guide future clinical trials to improve medical decision making, facilitate the use of MRI scanning, and stratify patients with brain injuries.

Other studies that have examined multiple biomarkers include those confirming proteomic trauma-release proteomes that identified a panel of astroglial injury biomarkers.^119^ A panel that covers different kinetic profiles and underlying processes is anticipated to improve TBI patient assessment over that of a single biomarker at a single time point.^209,210^ Some groups have introduced exploratory factor analysis to show significant commonalities among astroglial biomarkers based on their temporal profile in TBI patients. Interestingly, biomarkers that converged together also had the same cellular release behaviors in a human trauma culture model.^119^ Therefore, subjecting biomarker panels to a simple, unbiased machine-learning algorithm can help elucidate injury types beyond cell death. The National Institutes of Health (NIH) consortium Translational Outcomes Project in Neurotrauma (TOP-NT) correlates clinically used biomarkers tau, p-tau, NfL, GFAP, and ALDOC with structural, biochemical and functional MRI and establishes harmonized assay and imaging protocols. Novel TBI endophenotypes are identified by histopathophysiology, which also provides construct validity and can advise a candidate biomarker's context of use.

The relationship between neurological activity and its ensuing deficits from secondary injury processes after TBI is poorly understood. Multi-modal approaches are needed to bridge this gap. Such efforts are just starting to combine MRI and EEG with biofluid biomarkers and clinical outcomes in TBI patients.^211^ These proof-of-principle findings from a small cohort indicate that characteristic acute EEG spectra can predict secondary injury processes such as unfolding subcortical, thalamic nuclei atrophy that correlate with 6-month functional outcomes. Additional studies are needed to link conventional bedside EEG with blood-based biomarkers, specific brain region alterations, and overall recovery of TBI patients.

Another important contribution for successful use of biomarkers as surrogate end-points of TBI treatment, beyond combining biomarkers and multi-modal monitoring, is creating an optimal workflow of serial biomarkers. With decision-tree placement, highly sensitive, or acutely elevated biomarkers should be included up front, whereas highly specific, resource-intensive, or delayed elevated biomarkers could be used in a second stage to guide appropriate therapy or triage (see Fig. 1).

## Regulatory Considerations for Biomarkers

The development process for biomarkers should include sufficient validation using standardized clinical outcome measures. In addition, it must follow regulatory procedures for use in drug development. The FDA Qualification Process for Drug Development Tools helps ensure that results from using a biomarker can be reliable for specific interpretation and application in regulatory decisions involved with developing a drug.

Generally, biomarkers can be accepted by the FDA for use in therapeutic product development through two pathways. First, a drug developer may engage directly with the FDA during the drug development process to reach agreement on the use of a particular biomarker in a specific development program. More recently, the FDA has offered a second pathway in which a biomarker can be “qualified” for a particular context of use through the FDA's Biomarker Qualification Program. This process begins by defining the intended context of use, and then examining the evidence required for that context.^212,213^

Scientifically validated biomarkers have the potential of reducing the length, cost, and uncertainty of drug development by providing fast and reliable information on specific neurotrauma endophenotypes, including microstructural disruption such as fiber damage, cell death, or inflammation. Biomarker levels may thereby provide new information on the extent of injury. This outlines how biomarkers can augment and hasten the path to precision medicine.

## Conclusion

Biomarkers that enable researchers and clinicians to identify injury, assist in prognosis and decision making, and monitor clinical recovery are needed for precision healthcare following TBI. As promising neuroimaging, fluid-based, and physiological biomarkers still require additional development and validation, efforts are underway to determine which biomarkers have adequate specificity, sensitivity, feasibility, and ease of use. To enhance clinical relevance, we summarize our findings with five major takeaways and, as part of our roadmap, summarize the research gaps with tangible recommendations for next steps, which include specific action items (Table 2).

**Table 2.** Actionable Research Recommendations for Biomarker Development Studies in Traumatic Brain Injury (TBI)

Gap	Recommendation
A lack of large, systematic observational studies that longitudinally collect and analyze multi-modal candidate biomarkers through the course of injury	• Execute prospective studies to identify biomarkers (e.g., fluid, imaging, and genetic) that can monitor TBI sequelae for pharmacological or other therapeutic interventions being tested • Identify and validate underlying injury pathways that correspond to biomarker signatures to determine novel interventional methods • Study the time course of post-injury trajectories for biomarker data acquisition • Validate neuroimaging, physiological, and fluid biomarkers for subacute and chronic TBI patients • Integrate modalities and time points within the same patients and same time points to substantiate personalized diagnosis • Develop and validate a point-of-care solution and clinical tool(s) that will improve testing facilitation, portability, availability, and access • Develop new funding mechanisms or incorporate specific funding into existing mechanisms for analyzing new or existing data to benefit secondary data analyses across studies • Establish guidelines to standardize the frequency of data collection across the research spectrum
Direct comparisons against adequate normative data to enable harmonization across studies	• Build and grow a normative neuroimaging library to increase availability of normative data from healthy populations to substantiate precision diagnosis • Foster global team science via an open data sharing and analysis platform to accelerate insights. • Harmonize and standardize data across platforms and through data repositories to enable data acquisition quality control and make assays reproducible using the same procedures and calibrants
Identify validated and reliable multi-modal biomarker data to improve patient stratification to guide diagnosis, prognosis, and monitoring	• Catalyze a global TBI genome-wide association studies (GWAS) effort by leveraging existing data and samples, and centralize analysis. • Using existing data sets, facilitate larger-scale testing of available well-characterized biofluid and neuroimaging collections to ensure predictive ability across the injury spectrum • Develop an understanding for how biomarker panels and profiles could help guide meaningful indicators of improvement and patient management, which may inform diagnosis, prognosis, and selection of therapy

### Major takeaways

The five major takeaways from our findings are as follows.

- Evidence supports the utility of serum (protein) biomarkers for aiding the diagnosis of concussion.
- Other markers, such as miRNAs, SNPs, or PRSs, may be useful as prognostic biomarkers, and provide insights into the pathophysiology of recovery from TBIs.
- Longitudinal studies of biomarker levels in patients with life-compromising symptoms of mTBI will further biomarker use for patient characterization.
- Neuroimaging biomarkers have shown promise in the diagnosis, prognosis, and monitoring of TBI patients.
- Advanced neuroimaging techniques currently serve as powerful research tools; however, further development and normative data are required – and under development – to allow for these techniques to be used clinically for individual TBI patients.
